# Exploring Menstrual Hygiene Practices and Awareness of Menstrual Cups Among Nursing Professionals: A Cross-Sectional Survey

**DOI:** 10.7759/cureus.61179

**Published:** 2024-05-27

**Authors:** Medha Davile, Neha Gangane, Mubashshera F Khan, Shalini Dange, Shuchita Mundle

**Affiliations:** 1 Department of Obstetrics and Gynaecology, All India Institute of Medical Sciences, Nagpur, IND; 2 Department of Community Medicine, All India Institute of Medical Sciences, Nagpur, IND

**Keywords:** attitude & practices, knowledge attitude, sustainable menstrual products, health education & awareness, sanitary pads, menstrual cup, menstrual health hygiene

## Abstract

Background: Menstrual hygiene management poses significant challenges globally, with widespread reliance on disposable sanitary pads contributing to health risks and environmental degradation. Sustainable alternatives like menstrual cups offer promising solutions but face barriers to adoption, including myths and misconceptions. Educational interventions are crucial in promoting eco-friendly menstrual hygiene practices.

Material and methods: A descriptive cross-sectional survey was conducted at All India Institute of Medical Sciences (AIIMS), Nagpur (Central India) among nursing officers from January to February 2022. A structured questionnaire assessed the demographic characteristics, menstrual hygiene practices, knowledge of menstrual cups, and attitude toward their use.

Results: Of the 101 participants, the majority were between the age group of 25 and 40 years (71, 70.3%) and were educated up to a Bachelor of Science degree in nursing (79, 78.21%). Overwhelmingly, 97 (96.03%) of the nursing officers used non-biodegradable sanitary pads. Frequent need to change and staining were cited as common difficulties. Although 97 (96.04%) of the participants had heard of menstrual cups, knowledge gaps existed regarding suitability for nulliparous women, material composition, cost, and lifespan. Despite expressing a desire for better alternatives by 56 (55.4%) participants, only one (0.99%) reported current menstrual cup usage, with 60 (59.4%) participants indicating future readiness to use.

Conclusion: Discovering nurses' menstrual hygiene habits, this study sheds light on their reliance on disposable pads over menstrual cups. Improving the acceptability of menstrual cups hinges on addressing current knowledge gaps such as their feasibility for use by nulliparous women, ease of insertion, cost, and environmental sustainability. Despite hurdles like misinformation, participants show openness to embracing new options with the right guidance and education.

## Introduction

Menstruation, an intricate biological phenomenon [1], symbolizes the initiation of reproductive capability in females [2]. This natural process, while fundamental to the female lifecycle, presents a significant challenge in terms of managing hygiene and comfort during menstrual cycles. Across India, a staggering portion of menstruating individuals, estimated at approximately 241 million (roughly 77.5%), resort to locally or commercially produced disposable sanitary napkins [3]. According to the Ministry of Drinking Water and Sanitation, Government of India, one billion pads are used and disposed of each month, totaling 12 billion pads annually. Of these, 33% are buried, 28% are disposed of with routine waste, 28% are left in the open, and 28% are burned in the open. The use of superabsorbent polymers, non-biodegradable plastic, glue, and other materials in these pads prevents their decomposition for a minimum of 500 to 800 years, leading to long-term deterioration of water and soil quality. Additionally, blood-soiled menstrual absorbents provide an ideal culture medium for disease-causing pathogens. In contrast, menstrual cups significantly reduce both economic burden and menstrual waste, as one cup can last up to 10 years [4]. Babagoli et al. estimated the annual cost of menstrual cups to be $3,270 for 1,000 girls, compared to $24,000 for sanitary pads. Furthermore, the menstrual cup intervention was found to be cost-effective in improving health outcomes, with a cost of $2,300 per disability-adjusted life years averted [5].

Similarly, within the United Kingdom, the usage of menstrual hygiene products primarily comprises non-organic tampons and disposable pads [6]. Despite advancements in product design and availability, the prevalent cultural norms and market trends perpetuate the dominance of these conventional items. This trend extends globally, with tampons being the preferred choice in Western Europe and the United States, where an estimated 100 million women utilize them over their reproductive years, each averaging around 11,000 tampons in their lifetime [7]. Disposable pads, considered more culturally acceptable in certain regions, witness even greater usage, further exacerbating the environmental burden posed by menstrual waste [8]. According to a study by Babbar et al., several sociodemographic factors influence the use of modern menstrual products, such as menstrual cups and tampons. These factors include the level of education, place of residence (urban or rural), family income, socioeconomic status, freedom of movement, access to basic handwashing and sanitation facilities, access to toilets, social taboos against vaginal insertion, exposure to media, and use of mobile phones [9].

Addressing this environmental challenge necessitates a paradigm shift toward sustainable menstrual hygiene products. Reusable alternatives such as cloth pads, banana fiber pads, compostable sanitary pads, period panties, and menstrual cups offer promising solutions to mitigate the environmental impact of menstrual waste. Among these options, menstrual cups stand out as a safe, effective, and eco-friendly choice, with studies worldwide affirming their efficacy and safety [10-14].

However, despite the evident benefits, the uptake of menstrual cups remains relatively low, attributed in part to persistent myths and misconceptions surrounding their use. For instance, in a pilot study conducted by Tembo et al., concerns regarding the impact of menstrual cups on young women's virginity emerged as a barrier to adoption [13]. Female participants aged 16-24 years, attending the community-based sexual and reproductive health services were offered the Menstrual Health Hygiene (MHH) intervention, which included either a menstrual cup or reusable pads, analgesia, and menstrual health and hygiene education. There was higher uptake of reusable pads (88.0%) than menstrual cups (12.0%).

Additionally, the lack of awareness regarding the environmental ramifications of conventional menstrual products further compounds the issue, as highlighted in research by Peberdy et al. [15]. Most of the participants are not aware of the amount of plastic in disposable menstrual products. Higher awareness influences the choice of environmentally friendly products.

Disposable sanitary pads emerged as the predominant choice for menstrual hygiene management among respondents in a comprehensive pan-India study conducted by Youth Ki Awaz and the Water Supply and Sanitation Collaborative Council [16]. The diversity in usage between disposable sanitary pads and alternative menstrual hygiene products was striking, with figures ranging from a significant 71% to minimal usage below 5%, respectively.

In light of these challenges, initiatives aimed at promoting sustainable menstrual hygiene practices are paramount. Nursing professionals play a crucial role in healthcare education and are often at the forefront of promoting health and hygiene practices. Their unique position allows them to influence not only patients but the communities, regarding menstrual hygiene practices. The objective of the present study is to investigate the current menstrual hygiene practices among nursing professionals at All India Institute of Medical Sciences (AIIMS), Nagpur, and assess their awareness and usage patterns regarding menstrual cups as an alternative menstrual hygiene product. This will help to identify specific gaps in knowledge and misconceptions. The insight gained will enable us to design targeted educational interventions that empower nursing professionals with accurate information and dispel myths surrounding eco-friendly menstrual products.

## Materials and methods

This was a descriptive cross-sectional survey carried out in the Department of Obstetrics and Gynecology, AIIMS, Nagpur from January 2022 to February 2022. The study recruited the nursing officers posted in various departments of AIIMS, Nagpur. The questionnaire (Appendix) was created by the investigators by reviewing previous literature [17] and was modified based on the requirements of the study. The questionnaire was validated by one expert from community medicine and five subject experts (gynecologists). The internal consistency of items in the questionnaire was tested and it was acceptable (Cronbach's alpha = 0.7). The questionnaire was piloted on 10 randomly selected nursing officers and necessary changes were made to ensure the understanding of the study participants. This pretested self-administered structured questionnaire was then used to collect quantitative data. The data were collected by the investigator after obtaining prior consent.

The structured questionnaire consisted of four parts. Part I includes data on demographic characteristics like age, level of education, marital status, parity, and type of family. Part II includes six questions on the current usage of menstrual protection. Part III includes seven questions on knowledge about various aspects of menstrual cups, such as source of information, material used in production, cost of the product, and duration of use. Part IV includes two questions on attitude toward the current/future use of menstrual cups and readiness for the use of menstrual cups in the future. Positive responses were given a score of 1. Complete confidentiality and anonymity of the data were assured.

Sample size calculation

Based on the study by Divakar et al. [18], assuming 56% of the respondents would feel the menstrual cup is a better alternative, with 20% relative precision and 5% alpha error, the sample size was estimated to be 76. Considering the sensitive issue and non-response rate of 25%, a total of 101 participants were recruited.

Data management and statistical analysis

Quantitative data were analyzed using IBM SPSS software version 19.0 (IBM Corp., Armonk, NY) and presented in tables as percentages, frequencies, and proportions. The overall knowledge about menstruation was measured out of seven knowledge-specific questions. Each correct response earned one point while an incorrect response earned zero point. The total scores of the participants were calculated and used to determine their knowledge about the menstrual cups. Knowledge-specific questions were used to choose the percentage of participants having good and poor knowledge depending on the score generated. Those having a score of three and above were considered to have good knowledge about the menstrual cup.

The attitude was tested by two questions. The first question was regarding whether they are in search of a better alternative to the current menstrual protection method and if yes, which one they are looking for. The second question was regarding the attitude toward the current/future use of menstrual cups and the factors preventing its use like lack of knowledge, fear of insertion, and cost of the product. Positive and negative response toward current and future use of menstrual cups was given scores of 1 and 0, respectively.

## Results

A total of 101 participants were enrolled in this study, with their socio-demographic characteristics outlined in Table 1. The majority of participants (71, 70%) fell within the age group of 25-40 years with a mean (± SD) age of 26.47 (± 2.4) years. Of the participants, 79 (78.21%) possessed educational qualifications up to a Bachelor of Science degree in nursing. Among 39 (38.62%) married participants, 23 (58.97%) were primipara (had one child).

**Table 1:** Demographic characteristics of the participants (n = 101).

S. No.	Variable	Frequency (%)
1	Age group	
	Mean age of the participants = 26.47, SD = 2.4 (years)	
	18-24	30 (29.70%)
	25-40	71 (70.30%)
2	Educational qualification	
	General nursing and midwifery (GNM)	15 (14.85%)
	Bachelor of Science (BSc) Nursing	79 (78.21%)
	Master of Science (MSc) Nursing	7 (6.93%)
3	Marital status	
	Married	39 (38.62%)
	Unmarried	62 (61.38%)
4	Parity	
	Nullipara	85 (84.15%)
	Primipara	14 (13.86%)
	Multipara	2 (1.98%)
5	Type of family	
	Joint family	32 (31.68%)
	Nuclear family	69 (68.32%)

Table 2 illustrates the menstrual hygiene practices among the participants. An astounding 97 (96.03%) participants reported using sanitary pads. Considering the average age of menarche as 12 years, the average duration for which they were using sanitary pads was 13-14 years. Number of pads required by 56 (55.44%) participants was three per day. Disposal of pads typically involved wrapping the pads in newspaper before discarding them, a method employed by 92 (91.08%) participants. Of the remaining four participants, two reported using period panties, one reported using tampons, and one reported using a menstrual cup.

**Table 2:** Practices of participants regarding current menstrual protection method (n = 101).

S. No.	Menstrual practices (n = 101)	Frequency (%)
1	Menstrual protection used in the last cycle	
	Sanitary pad	97 (96.03%)
	Tampons	1 (0.99%)
	Cloth	0 (0%)
	Menstrual cup	1 (0.99%)
	Period panty	2 (1.98%)
2	Frequency of changing the sanitary products	
	Once	1 (0.99%)
	Twice	33 (32.68%)
	Thrice	56 (55.44%)
	More than thrice	11 (10.89%)
3	Current practice of disposing of sanitary products	
	Throw in dustbin	4 (3.96%)
	Wrap in newspaper and throw in dustbin	92 (91.08%)
	Burn	2 (1.98%)
	Store to reuse	2 (1.98%)
	Wrap in newspaper and burn	1 (0.99%)
4	Difficulties faced while using current menstrual protection	
	Stock out of sanitary product	6 (5.94%)
	Need to change frequently	35 (34.65%)
	Fear of staining	23 (22.77%)
	Stinking	22 (21.78%)
	Need to wash and reuse	3 (2.97%)
	Cost	6 (5.94%)
	Difficulty in disposal	15 (14.85%)
	No difficulty	30 (29.70%)

The reported difficulties with the most commonly used current menstrual protection included the need for frequent changes (35, 34.65%), concerns about staining (23, 22.77%), especially during the night, and odor (22, 21.78%).

In assessing participants' knowledge of menstrual cups, inquiries were posed regarding familiarity, suitability for nulliparous women, cost, and materials (Table 3). Participants were categorized based on their knowledge scores, with 71 (70.3%) deemed to have insufficient knowledge about menstrual cups. While 97 (96.04%) participants had heard of menstrual cups, around 82 (81.18%) believed they could be used by nulliparous women. Additionally, 73 (72.27%) were unaware of the cup's material composition, and a mere seven (6.93%) knew of its 10-year lifespan. Almost half (47, 46.53%) of the participants were unfamiliar with its cost.

**Table 3:** Knowledge regarding menstrual cup (n = 101).

S. No.	Knowledge regarding menstrual cups	Frequency (%)
1	Have you ever heard about the menstrual cup?	
	Yes	97 (96.04%)
	No	4 (3.96%)
2	How long a menstrual cup can be used in the menstrual cycle?	
	Six months	33 (32.67%)
	One year	24 (23.76%)
	Five years	13 (12.87%)
	10 years	7 (6.93%)
	Don’t know	24 (23.76%)
3	Can it be used in nulliparous women?	
	Yes	82 (81.18%)
	No	14 (13.86%)
	Don’t know	5 (4.95%)
4	Do you have any information about the material used in making menstrual cups?	
	Yes	28 (27.72%)
	No	73 (72.27%)
5	Do you have any idea about the cost of menstrual cups?	
	I do not have any idea	47 (46.53%)
	Costlier than regular sanitary pads	18 (17.82%)
	Cheaper than regular sanitary pads	33 (32.67%)
	Expenditure will be equal in both	3 (2.97%)

Interestingly, 56 (55.4%) expressed a desire for better alternatives to their current menstrual protection, with 60 (59.4%) indicating readiness to consider menstrual cups in the future.

In-depth interviews were conducted with 12 participants who were using sanitary pads to explore their views on current menstrual product usage and attitudes toward menstrual cups. Common challenges cited included the fear of staining and the inconvenience of frequent changes during heavy flow days, particularly in workplace settings where privacy is limited. Difficulty in the disposal of sanitary pads at the workplace was attributable to limited privacy. Lack of knowledge about proper insertion and removal methods was quoted as the primary reason for not currently using menstrual cups, with many expressing apprehension about the insertion.

Among the two participants who were using period panties, one had been using them for the last two years, and the other one for the last eight months. Both were using sanitary pads before shifting to the current menstrual product. Washing with soap and water is the method employed by these participants. One participant has been using menstrual cups for the last one and a half years after using sanitary pads for years. She needs to empty it twice a day and clean it with plain water after emptying it. One more participant has been using tampons for the last one year. Lack of leakage and suitability to use at the workplace are the advantages cited by these participants.

## Discussion

This descriptive cross-sectional study delved into the prevalent practices of menstrual hygiene among nursing officers. It revealed that a staggering 96.03% relied on non-biodegradable sanitary pads. These findings echo similar trends observed in studies among college-going women done by Durairaj et al., reaffirming the dominance of sanitary pads as the preferred method of menstrual protection [14]. Despite the growing dialogue on modern alternatives like menstrual cups and tampons, their adoption remains dismally low, with only one participant reporting the use of a menstrual cup in the present study - a trend consistent with existing literature.

Numerous studies have underscored the popularity of sanitary pads among Indian women, citing factors such as ease of use, accessibility, and affordability [19,20]. Recent National Family Health Survey-5 (NFHS-5, 2019-21) further corroborates this, with minimal usage reported for menstrual cups (0.3%) and tampons (1.57%) among young menstruating individuals [3]. Similarly, cross-sectional studies from specific regions like Gujarat shed light on the overwhelming preference for sanitary pads (96.06%) over modern alternatives [21]. Similarly, in a study by Choi et al., the most common types of menstrual hygiene products across all age groups were disposable menstrual pads (89.0%), followed by cloth menstrual pads (4.5%), tampons (4.2%), and only 1.6% used a menstrual cup [22].

Insights from studies conducted in diverse settings reveal both barriers and facilitators to the uptake of menstrual cups. In Kerala, in a study by Sudevan Devan et al. [17], concerns regarding insertion fear and discomfort emerged as predominant deterrents, while in a study by Pokhrel et al. in Nepal [23], schoolgirls expressed positive perceptions of menstrual cups, citing benefits such as improved school attendance and environmental sustainability. However, common discomforts like pain during insertion and leakage were noted across contexts, indicating the need for comprehensive education on cup usage.

Mason et al. [24] conducted a study comparing the acceptance of menstrual cups and sanitary pads among rural schoolgirls in western Kenya. Initially, participants faced challenges with inserting, removing, and finding comfort with menstrual cups, but these difficulties diminished over time. Conversely, sanitary pads were more readily accepted, with fewer reported issues. This depicts the significance of consistent practice, which can enhance the acceptance of menstrual cups.

The strength of the study is that it includes a detailed demographic analysis of 101 participants. By identifying the common difficulties associated with current menstrual products, such as frequent changes, staining, and odor, the study highlights the practical issues faced by women, which can guide future interventions. The study thoroughly assesses participants' knowledge about menstrual cups, revealing significant gaps in awareness and understanding. This highlights the need for educational interventions and provides a clear direction for future educational efforts. The finding that over half of the participants expressed a desire for better menstrual protection alternatives and a willingness to consider menstrual cups in the future indicates a potential receptiveness to change, which is crucial for designing effective interventions.

Educational interventions, such as instructional videos depicting complete information about the cup, including the material used for making the cups, the volume of the cup, how to insert, frequency of emptying, lifespan of the cup, and health benefits, hold promise in fostering informed decision-making. Additionally, highlighting the long-term economic and environmental advantages of menstrual cups can further incentivize their use over disposable alternatives.

Limitations

The discussion of this study brings to light several critical limitations that merit consideration. Firstly, the study participants included nursing professionals and this narrow demographic scope may limit the generalizability of the findings to broader populations of menstruating individuals. Additionally, the reliance on self-reported data introduces the potential for recall bias and subjective interpretation, which may impact the accuracy of the results.

Though the study sheds light on the limited adoption of menstrual cups among nursing officers, it does not delve deeply into the underlying reasons for these patterns. Exploring the socio-cultural, economic, and educational factors influencing menstrual hygiene practices could offer valuable insights into strategies for promoting the uptake of eco-friendly alternatives.

Scope for further research

Multicenter studies based on the actual use of sustainable menstrual products and dissemination of the results depicting its benefits would be more impressive for acceptance of these products among eligible girls and women.

## Conclusions

In conclusion, this study offers valuable insights into the menstrual hygiene practices and perceptions of nursing officers, highlighting the prevalent use of disposable sanitary pads and the limited uptake of menstrual cups. Despite facing challenges such as incomplete knowledge and misconceptions, the participants expressed a willingness to explore alternative menstrual hygiene options if provided with accurate information.

As champions for women's health, nursing officers are well-positioned to lead the efforts and serve as catalysts for positive change in menstrual hygiene management practices. Ultimately, it can contribute to environmental sustainability.

## Figures and Tables

**Figure 1:**
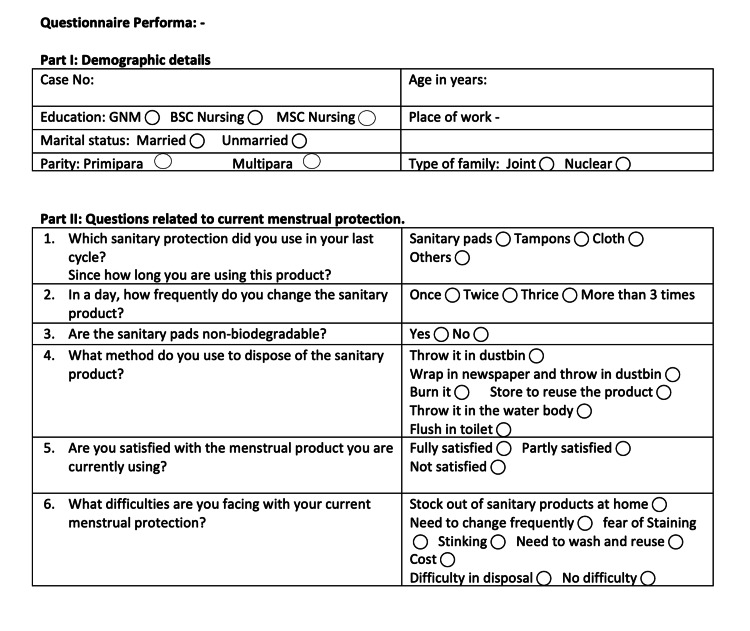
Questionnaire - Part I and II.

**Figure 2:**
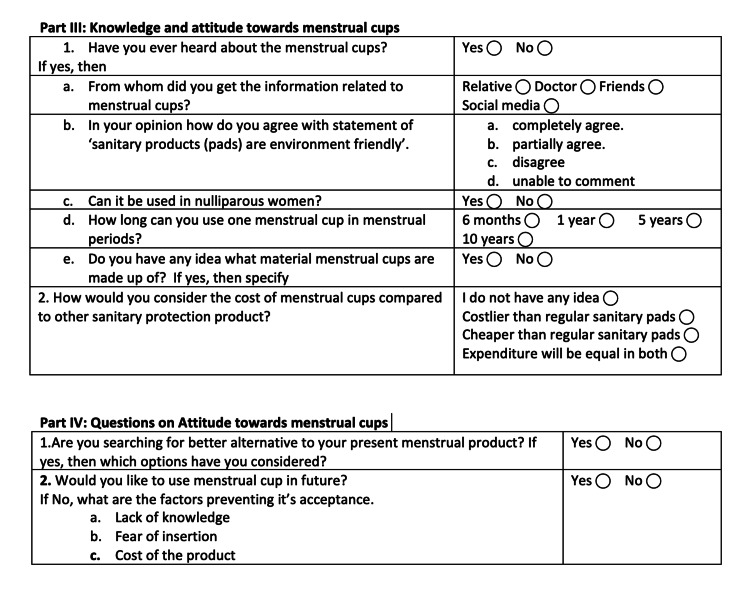
Questionnaire - Part III and IV.

## Appendices

Questionnaire
